# Cultivation of Acidophilic Algae *Galdieria sulphuraria* and *Pseudochlorella* sp. YKT1 in Media Derived from Acidic Hot Springs

**DOI:** 10.3389/fmicb.2016.02022

**Published:** 2016-12-20

**Authors:** Shunsuke Hirooka, Shin-ya Miyagishima

**Affiliations:** ^1^Department of Cell Genetics, National Institute of GeneticsMishima, Japan; ^2^Japan Science and Technology Agency, Core Research for Evolutionary Science and TechnologyKawaguchi, Japan; ^3^Department of Genetics, Graduate University for Advanced StudiesMishima, Japan

**Keywords:** acidic hot spring water, phycocyanin, lipid droplets, nitrogen source, phosphorus, biomass

## Abstract

Microalgae possess a high potential for producing pigments, antioxidants, and lipophilic compounds for industrial applications. However, the cultivation of microalgae comes at a high cost. To reduce the cost, changes from a closed bioreactor to open pond system and from a synthetic medium to environmental or wastewater-based medium are being sought. However, the use of open pond systems is currently limited because of contamination by undesirable organisms. To overcome this issue, one strategy is to combine acidophilic algae and acidic drainage in which other organisms are unable to thrive. Here, we tested waters from sulfuric acidic hot springs (Tamagawa, pH 1.15 and Tsukahara, pH 1.14) in Japan for the cultivation of the red alga *Galdieria sulphuraria* 074G and the green alga *Pseudochlorella* sp. YKT1. Both of these spring waters are rich in phosphate (0.043 and 0.145 mM, respectively) compared to other environmental freshwater sources. Neither alga grew in the spring water but they grew very well when the waters were supplemented with an inorganic nitrogen source. The algal yields were ∼2.73 g dry weight/L for *G. sulphuraria* and ∼2.49 g dry weight/L for *P*. sp. YKT1, which were comparable to those in an autotrophic synthetic medium. *P*. sp. YKT1 grew in the spring waters supplemented either of NH_4_^+^, NO_3_^-^ or urea, while *G. sulphuraria* grew only when NH_4_^+^ was supplemented. For *P*. sp. YKT1, the spring water was adjusted to pH 2.0, while for *G. sulphuraria*, no pH adjustment was required. In both cases, no additional pH-buffering compound was required. The phycocyanin of the thermophilic *G. sulphuraria* is known to be more thermostable than that from the *Spirulina platensis* currently used in phycocyanin production for commercial use. The phycocyanin content in *G. sulphuraria* in the Tsukahara water supplemented with NH_4_^+^ was 107.42 ± 1.81 μg/mg dry weight, which is comparable to the level in *S. platensis* (148.3 μg/mg dry weight). *P*. sp. YKT1 cells in the Tamagawa water supplemented with a nitrogen source formed a large amount of lipid droplets while maintaining cellular growth. These results indicate the potential of sulfuric hot spring waters for large-scale algal cultivation at a low cost.

## Introduction

Microalgae have great potential as a source of pigments, antioxidants, lipophilic compounds and biofuels for industrial applications (Milledge, 2012). For example, they contain phycobilins (Singh et al., 2005), carotenoids (Borowitzka, 2010) and long-chain polyunsaturated fatty acids (Abedi and Sahari, 2014). However, large-scale cultivation of microalgae for commercial use is still costly, so has remained limited to the production of relatively expensive materials. For algal cultivation, closed bioreactors with a synthetic medium are optimal and have often been adopted to maximize algal growth and/or the production of desirable compounds. However, the cost to build and maintain the reactors as well as the necessary water and chemicals is relatively expensive. Thus, open pond culture systems as well as natural and/or wastewater are alternative means for reducing the cost of microalgal cultivation. The major problem in open pond systems is that the culture is easily contaminated by undesirable microorganisms, including algal predators. In addition, the challenge in using natural water is to avoid impinging upon agriculture and domestic use.

One of the strategies to overcome the problem of contamination in open pond systems is to utilize environments in which only the organism of interest is tolerated. Thus far, open pond cultivation has been applied for commercial use in limited algal species, such as *Spirulina*, *Chlorella*, and *Dunaliella* spp. (Borowitzka, 1999) in certain environments (e.g., high alkaline or high salt) that are lethal for other organisms.

In a highly acidic environment, the number of species is very limited compared to a neutrophilic environment, but a number of acidophilic bacteria, archaea and eukaryotes have been identified, mainly by community structure analyses (López-Archilla et al., 2001; Amaral-Zettler et al., 2002). Among the eukaryotes, acidophilic microalgae such as the red and green algae have been reported to exist in a relatively higher abundance (Aguilera et al., 2007, 2010). In addition, acidic wastewater is easily available from acidic hot springs and mines and, in many cases, drainage are processed so as to be neutralized and detoxified before release into the environment by defraying the cost. Thus, the combination of acidophilic algae in acidic natural wastewater will be one strategy applied to open pond culture systems for commercial use or bioremediation in the future.

To this end, in this study, we tested water from two Japanese sulfuric acidic hot springs (Tamagawa and Tsukahara; pH ∼1) for cultivation of acidophilic algae. Sulfuric acidic hot springs have been reported worldwide, such as Yellowstone in USA and several springs in New Zealand, Italy (Stan-Lotter and Fendrihan, 2012). As representative acidophilic algae, we tested the red alga *Galdieria sulphuraria* 074G (Gross and Schnarrenberger, 1995) and the green alga *Pseudochlorella* sp. YKT1 (Hirooka et al., 2014).

*Galdieria sulphuraria* belongs to the Cyanidiophyceae which is the dominant form in sulfuric acidic hot springs worldwide (pH 0.05–5.0, 35–56°C; but they are able to grow slowly at lower temperatures, for example at 20°C) (Reeb and Bhattacharya, 2010). Red algae contain phycocyanin (blue pigment) for photosynthesis, as in the case of cyanobacteria, a pigment which is commonly used in cosmetics, diagnostics and foods as well as nutraceutical and biopharmaceutical products (Borowitzka, 2013).

In addition, the Cyanidiophyceae are tolerant to heavy metals and absorb high concentrations of them from an aquatic environment (Nagasaka et al., 2004; Misumi et al., 2008). This is because metals are easily ionized and dissolved in acidic water. Recently, *G. sulphuraria* was shown to selectively recover rare earth elements (Minoda et al., 2015). It is reported that Tamagawa hot spring contains rare earth elements (Sanada et al., 2006). Thus, the cultivation of *G. sulphuraria* in acidic hot spring water will be applied to efforts to concentrate rare metals.

The green alga *Pseudochlorella* sp. YKT1 (Trebouxiophyceae) was recently isolated from the acidic drainage of an abandoned sulfur mine in Japan (Hirooka et al., 2014). This alga is able to grow at pH 2.0–5.0 and 20–32°C and accumulates a large amount of storage lipids (∼30% of dry weight) under a nitrogen-depleted condition, a characteristic which is likely useful for the production of biofuels.

Here we show that both *G. sulphuraria* and *P.* sp. YKT1 grow well when the spring water is supplemented with an inorganic nitrogen source. In addition, *G. sulphuraria* and *P.* sp. YKT1 have different preferences in terms of the nitrogen source. Our results suggest that environmental acidic wastewater will be useful to reduce the cost of the medium used for algal cultivation and open pond systems.

## Materials and Methods

### Measurement of the Ammonium, Nitrate and Phosphorus Concentrations in Acidic Hot Spring Water

Acidic water was collected from the Tsukahara (Yufu, Oita prefecture) and Tamagawa (Senboku, Akita prefecture) sulfuric hot springs in Japan. To determine the ammonium and nitrate concentrations, the spring water (25 mL) was adjusted to pH 7.0 with NaOH, and diluted to 50 mL with distilled water. The water sample was centrifuged (2,000 × *g* for 5 min) and the supernatant fraction was transferred into a new tube.

The ammonium concentration was determined by the indophenol method of Scheiner (1976). Five hundred microliter of the neutralized water sample were transferred into a 1.5 mL tube, then 200 μL of phenol nitroprusside solution [60 mg/mL phenol, 0.2 mg/mL Na_2_[Fe(CN)_5_NO]⋅2H_2_O, diluted to 100 mL with buffer (30 mg/mL Na_3_PO_4_⋅12H_2_O, 30 mg/mL Na_3_C_6_H_5_O_7_⋅2H_2_O, 3 mg/mL EDTA)] and 300 μL of 0.08–0.11 w/v% sodium hypochlorite solution were added, with thorough mixing performed after each addition. After incubation at 30°C for 45 min, the absorbance at 635 nm was measured with a spectrophotometer (SmartSpec Plus; BIO-RAD, Richmond, CA, USA). Ammonium chloride stock solution (0.01 mg/mL in distilled water) was used as the NH_4_^+^ standard (in a range from 0.01 to 0.3 mg/L) to obtain a calibration curve.

The nitrate concentration was determined by the brucine-sulfanilic method of Jenkins and Medsker (1964). Hundred microliter of the neutralized water sample was transferred into a 1.5 mL tube, then 50 μL of brucine-sulfanilic acid solution (10 mg/mL brucine dihydrate, 1 mg/mL sulfanic acid, and 30 μL/mL hydrogen chloride), 500 μl of sulfuric acid solution (sulfuric acid: water = 20: 3) and 500 μL of distilled water were added, with thorough mixing after each addition. After incubation at 4°C for 45 min, the absorbance at 410 nm was measured with the spectrophotometer. Potassium nitrate stock solution (0.1 mg/mL in distilled water) was used as the NO_3_^-^ standard (in a range from 0.02 to 1.0 mg/L) to obtain a calibration curve.

The phosphate concentration was determined by the colorimetric method according to Murphy and Riley (1962). Eight hundred microliter of the natural water sample was transferred into a 1.5 mL tube, then 160 μL of antimony-molybdate solution (1.25 M sulfuric acid, 30 mM ascorbic acid, 0.13715 mg/mL antimony potassium tartrate, and 60 mg/mL ammonium molybdate tetrahydrate) and 40 μL of distilled water were added, with thorough mixing after each addition. After incubation at 30°C for 20 min, the absorbance at 710 nm was measured with the spectrophotometer. Potassium dihydrogen phosphate stock solution (0.005 mg/mL in distilled water) was used as the PO_4_^3-^ standard (in a range from 1.0 to 4.0 mg/L) to obtain a calibration curve.

### Algal Strains

The red alga *G. sulphuraria* strain 074G (Gross and Schnarrenberger, 1995) and *Pseudochlorella* sp. YKT1 (Hirooka et al., 2014) were used in this study. *G. sulphuraria* was maintained with gyration in M-Allen (MA) medium (Minoda et al., 2004), at pH 2.0 and 30°C under continuous light. *P.* sp. YKT1 was maintained with gyration in MA medium at pH 2.5 and 21°C under continuous light.

### Culture Media and Conditions

Two natural acidic water samples from Tamagawa (Ta; pH 1.15) and Tsukahara (Tsu; pH 1.14) were used to prepare the cultivation media. To produce Ta + NH_4_^+^, Tsu + NH_4_^+^, Ta + NO_3_^-^, Tsu + NO_3_^-^, Ta + urea or Tsu + urea, 10 mM (NH_4_)_2_SO_4_, 20 mM NaNO_3_ or 10 mM urea were supplemented to Ta and Tsu, respectively. To produce the Ta + P and Tsu + P media, 2 mM KH_2_PO_4_ were supplemented to Ta and Tsu, respectively. To produce Ta + NH_4_^+^P or Tsu + NH_4_^+^ P, 10 mM (NH_4_)_2_SO_4_ and 2 mM KH_2_PO_4_ were supplemented to Ta and Tsu, respectively. Each medium was filter-sterilized (the pore size was 0.22 μm). As a synthetic media, either the MA medium or nitrogen-free MA medium (MA-N; the 10 mM (NH_4_)_2_SO_4_ was replaced by 10 mM NaSO_4_) at pH 2.0 was used.

The *G. sulphuraria* cells cultured in MA medium at 40°C (OD_750_ of 1.0–2.0) were collected by centrifugation at 1,500 × *g* for 5 min and then gently resuspended into each medium to give an OD_750_ of 1.0. After resuspension into 30 mL of medium in a 100 mL test tube, cells were cultured at 40°C under continuous light (90 μE/m^2^⋅s) with aeration (0.3 L ambient air/min).

For cultivation of *P.* sp. YKT1, all of the media derived from the natural acidic water were adjusted to pH 2.0 with KOH. *P.* sp. YKT1 cells cultured in MA medium at 21°C (OD_750_ of 1.0–2.0) were collected by centrifugation at 1,500 × *g* for 5 min and then gently resuspended into each medium to give an OD_750_ of 1.0. Cells were cultured with gyration in a 24-well culture plate or Erlenmeyer flasks at 25°C under continuous light (90 μE/m^2^⋅s).

### Determination of the Chlorophyll and Phycocyanin Contents along with the Dry Weight

The chlorophyll and phycocyanin contents were determined by spectrophotometric method according to Misumi et al. (2016). Briefly, the cell culture was diluted with fresh medium to cell density of OD_750_ = 0.5. Then absorbance was measured at wavelengths of 620 and 678 nm in a cuvette with a light path length of 10 mm by a spectrophotometer (UV-2600; Shimazu, Kyoto, Japan) equipped with an integrating sphere (ISR-2600Plus; Shimazu, Kyoto, Japan). The chlorophyll and phycocyanin contents were calculated as [Chl a] = 14.97 × A_678_ – 0.615 × A_620_ and [PC] = 138.5 × A_620_ – 35.49 × A_678_ according to Arnon et al. (1974). For dry weight determination, cell cultures were filtered using a pre-weighed 0.45 μm HA MF-MILLIPORE MEMBRANE (Millipore Corp., Bedford, MA, USA). The membrane was dried at 50°C for 2 h and weighed on a microbalance.

### BODIPY Staining and Fluorescence Microscopy

Cellular neutral lipids were stained with the fluorescent dye dipyrrometheneboron difluoride (BODIPY) according to the method of Kuroiwa et al. (2012) with minor modifications. Briefly, 95 μL of the cell suspension were stained with 5 μL of 10 μM BODIPY stock solution. The stained samples were observed under epifluorescence microscope (BX51; Olympus, Tokyo, Japan) fit with a digital camera (DP71; Olympus, Tokyo, Japan) under green excitation (for chloroplast autofluorescence) or blue excitation (for BODIPY fluorescence). Images were processed digitally with Photoshop software (Adobe Systems, Mountain View, CA, USA).

## Results

### Nitrogen and Phosphorus Concentrations in Acidic Hot Spring Water

Hot spring water samples from Tamagawa and Tsukahara hot springs in Japan were tested for their applicability to algal cultivation. The pH of Ta and Tsu were 1.15 and 1.14, respectively (**Table 1**). Because inorganic nitrogen and phosphorus sources limit the growth of algae in natural environments (Hecky and Kilham, 1988), we first measured the inorganic nitrogen (NH_4_^+^ and NO_3_^-^) and phosphorus (PO_4_^3-^) concentrations in Ta and Tsu (**Table 1**).

**Table 1:** pH, inorganic nitrogen (NH_4_^+^ and NO_3_^-^) and phosphorus (PO_4_^3-^) concentrations in the Ta and Tsu hot spring waters and synthetic MA medium.

	pH	NH_4_^+^ concentration	NO_3_^-^ concentration	PO_4_^3-^ concentration
		(mM)	(mM)	(mM)
Ta	1.15	0.0021	ND	0.043
Tsu	1.14	0.0086	ND	0.145
MA	2.0	10	–	2

The ammonium (NH_4_^+^) concentration in Ta and Tsu was 0.0021 mM (0.038 mg/L) and 0.0086 mM (0.156 mg/L), respectively. The nitrate (NO_3_^-^) concentration in Ta and Tsu was below the detection limit. The phosphorus (PO_4_^3-^) concentration in Ta and Tsu was 0.043 mM (4.07 mg/L) and 0.145 mM (13.75 mg/L), respectively. Thus, the total inorganic nitrogen level was in the range of an oligotrophic lake (<0.35 mg/L in total nitrogen), while the phosphorus concentration was in the range of a eutrophic lake (>0.035 mg/L in total phosphorus) (Galvez-Cloutier and Sanchez, 2007).

### The Growth of *G. sulphuraria* in Media derived from Acidic Hot Spring Water

We then investigated whether the red alga *G. sulphuraria* strain 074G (Gross and Schnarrenberger, 1995) would grow in the media derived from acidic hot spring water samples under laboratory conditions. First, we prepared Ta and Tsu water as they were prepared above, i.e., Ta + NH_4_^+^ and Tsu + NH_4_^+^ (supplemented with 10 mM NH_4_^+^), Ta + P and Tsu + P (supplemented with 2 mM PO_4_^3-^), and Ta + NH_4_^+^ P and Tsu + NH_4_^+^ P (supplemented with 10 mM NH_4_^+^ and 2 mM PO_4_^3-^) (all filter-sterilized) to test for algal growth. The levels of the additional NH_4_^+^ and PO_4_^3-^ were the same as that in the synthetic inorganic MA medium, which is suitable for growth of Cyanidiophyceae red algae (Minoda et al., 2004) and much higher than the original level in the spring water (**Table 1**). For comparison, an inorganic synthetic medium for autotrophic growth (MA) and a nitrogen-depleted MA (MA-N) medium were also used.

**FIGURE 1:**
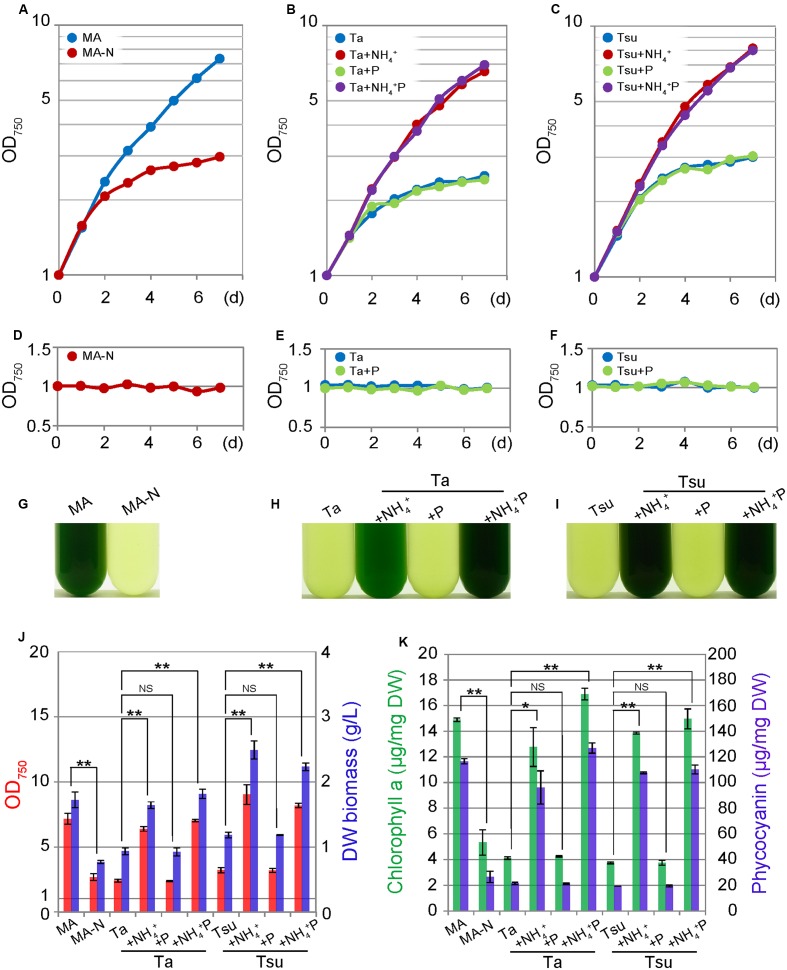
**Growth of *G. sulphuraria* 074G cells in synthetic MA, nitrogen-depleted MA-N and media derived from acidic hot spring waters. (A–C)** Change in the OD_750_ of the culture in the respective media. Cells cultured in synthetic MA medium (with an OD_750_ of 1.0–2.0) were collected by centrifugation, resuspended into the respective media to give an OD_750_ of 1.0 and then cultured for 7 days. **(D–F)** The change in the OD_750_ of the culture in the respective media. Cells cultured for 7 days in each medium were again inoculated into the same medium. **(G–I)** Photographs of the cultures in the respective media at 7 days after inoculation. **(J)** OD_750_ (red bar) and algal dry weight (DW biomass; blue bar) at 7 days after inoculation into the respective media after being taken from the MA medium. **(K)** Chlorophyll *a* (green bar) and phycocyanin (blue bar) contents per algal dry weight (DW) at 7 days after inoculation into the respective media after being taken from the MA medium. Three independent experiments showed similar results and the results from one experiment are shown **(A–I)**. The bar indicates the standard deviation of three independent experiments **(J,K)**. Significance was calculated by *t*-test. ^∗^Statistically significant differences at *P* < 0.05; ^∗∗^statistically significant differences at *P* < 0.01; NS, not significant **(J,K)**.

In the media derived from spring water, in which NH_4_^+^ was not supplemented (spring water media other than +NH_4_^+^ and +NH_4_^+^ P), *G. sulphuraria* did not grow (**Figures 1H,I**), as was also the case for the nitrogen-depleted synthetic medium MA-N (**Figure 1G**). However, in spring water media other than +NH_4_^+^ and +NH_4_^+^ P, the OD_750_ value slightly increased after inoculation (**Figures 1B,C**), the OD_750_ value never increased when the cells were again inoculated into the same medium (**Figures 1E,F**).

**FIGURE 2:**
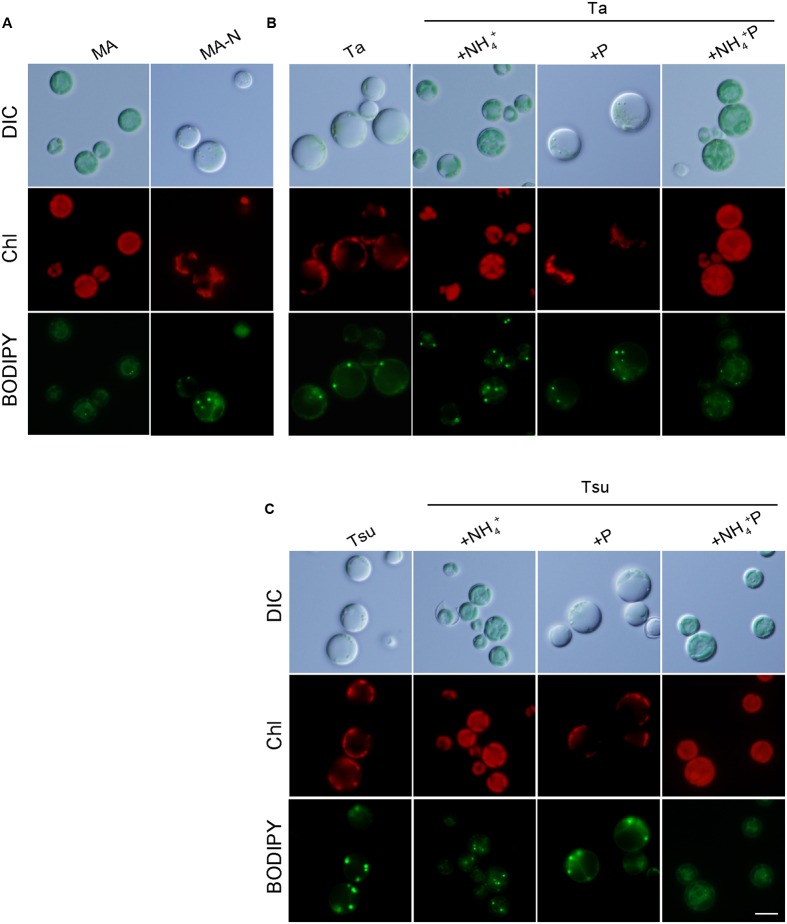
**Microscopic observation of *G. sulphuraria* 074G cells in MA, MA-N and media derived from acidic hot spring waters. (A–C)** Micrographs of cells that were cultured in the respective media. Cells cultured in synthetic MA medium (OD_750_ of 1.0–2.0) were collected by centrifugation, resuspended into the respective media to give an OD_750_ of 1.0 and then cultured for 7 days. Images obtained by differential interference contrast microscopy (DIC), autofluorescence of chloroplasts (Chl) and BODIPY staining (BODIPY) are shown. Scale bar = 5 μm.

After inoculation into spring water media other than the +NH_4_^+^ and +NH_4_^+^P, the cellular chlorophyll and phycocyanin contents decreased compared with those in MA, as in the case of MA-N (**Figure 1K**). These decreases were also evident under microscopy, in which green chloroplasts were severely decreased in total area, while the red autofluorescence of the photosynthetic pigments was also decreased in the spring water media other than +NH_4_^+^ and +NH_4_^+^ P as well as MA-N (**Figure 2**). The degradation of the phycobilisome as a major store of cellular nitrogen and reduction in size of the chloroplasts are known to occur upon nitrogen starvation in cyanobacteria and eukaryotic algae (Görl et al., 1998; Sinetova et al., 2006). Thus, the initial slight increase of the OD_750_ in spring water media other than +NH_4_^+^ and NH_4_^+^ P (**Figures 1B,C**) as well as MA-N (**Figure 1A**) was probably due to the degradation of phycobilisomes and other proteins, not autotrophic growth.

In contrast to above results, cells grew well in spring water media in which the nitrogen source was supplemented (+NH_4_^+^ and +NH_4_^+^ P media) in a time course and at a concentration similar to cells cultured in the synthetic MA medium (**Figures 1A–I**). The cellular chlorophyll and phycocyanin contents in the cells in the +NH_4_^+^ and +NH_4_^+^P media were comparable to those in MA (**Figure 1K**), and the cells maintained green chloroplasts, the size of which was similar to that in the cells in the synthetic MA medium (**Figure 2**).

Because algae are considered a potential source of next-generation biofuels, we observed lipid droplet formation in the cells cultured in spring water media by means of BODIPY staining (**Figure 2**). In the synthetic MA medium, lipid droplets were scarcely detected in the cells (**Figure 2A**). Eukaryotic algae are known to accumulate triacylglycerol in lipid droplets under a nitrogen-starved condition. Consistent with previous reports in other eukaryotic algae (e.g., Wang et al., 2009; Kuroiwa et al., 2012, 2014; Simionato et al., 2013), in spring water media without any additional nitrogen source (Ta, Tsu, Ta + P, and Tsu + P), as well as in MA-N, some lipid droplets were formed in the cells along with a reduction of the chloroplasts and inhibition of growth (**Figure 2**). In contrast with the nitrogen-depleted or otherwise limited conditions described above, we also observed lipid droplet formation in the spring water media in which additional nitrogen but not phosphate was supplemented (Ta + NH_4_^+^ and Tsu + NH_4_^+^), in which cells grow without any decrease in the chloroplasts (**Figure 2**).

### The Growth of *G. sulphuraria* Using Different Nitrogen Sources

**FIGURE 3:**
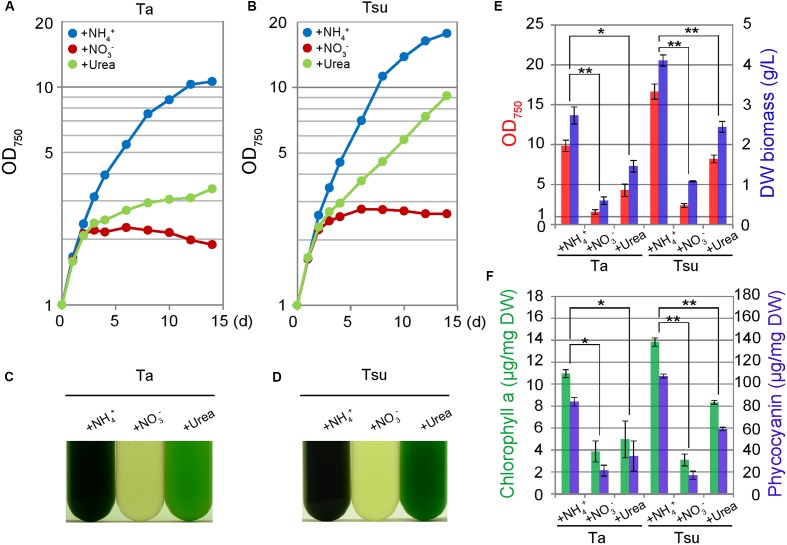
**Growth of *G. sulphuraria* cells in media derived from acidic hot spring waters with different nitrogen sources**. Cells cultured in synthetic MA medium (OD_750_ of 1.0–2.0) were collected by centrifugation, resuspended into the respective media to give an OD_750_ of 1.0 and then cultured for 14 days. **(A–D)** Change in OD_750_ **(A,B)** and photographs **(C,D)** of the cultures in the respective media. Photographs were taken 14 days after the inoculation. **(E,F)** OD_750_ (red bar), algal dry weight (DW biomass; blue bar) **(E)** chlorophyll *a* (green bars) and phycocyanin (blue bar) contents per algal dry weight **(F)** at 14 days after the inoculation. Three independent experiments showed similar results and the results from one experiment are shown **(A–D)**. The bars indicate the standard deviation of three indipendent experiments **(E,F)**. Significance was calculated by *t*-test. ^∗^Statistically significant differences at *P* < 0.05; ^∗∗^Statistically significant differences at *P* < 0.01 **(E,F)**.

The above results show that an additional inorganic nitrogen source is essential to culture *G. sulphuraria* in acidic hot spring waters. Three different nitrogen sources were tested for cultivation of this alga (10 mM (NH_4_)_2_SO_4_, 20 mM NaNO_3_ or 10 mM urea) (**Figure 3**). Among the three nitrogen sources, ammonium proved to be the most favorable for cellular growth (**Figure 3**). The culture entered into a stationary phase 14 days after inoculation (**Figures 3A,B**) and the dry weight biomass reached 2.73 ± 0.21 and 4.11 ± 0.14 g/L in Ta + NH_4_^+^ and Tsu + NH_4_^+^, respectively (**Figure 3E**). The phycocyanin content reached 84.12 ± 3.93 and 107.42 ± 1.81 μg/mg dry weight in Ta + NH_4_^+^ and Tsu + NH_4_^+^, respectively (**Figure 3F**).

**FIGURE 4:**
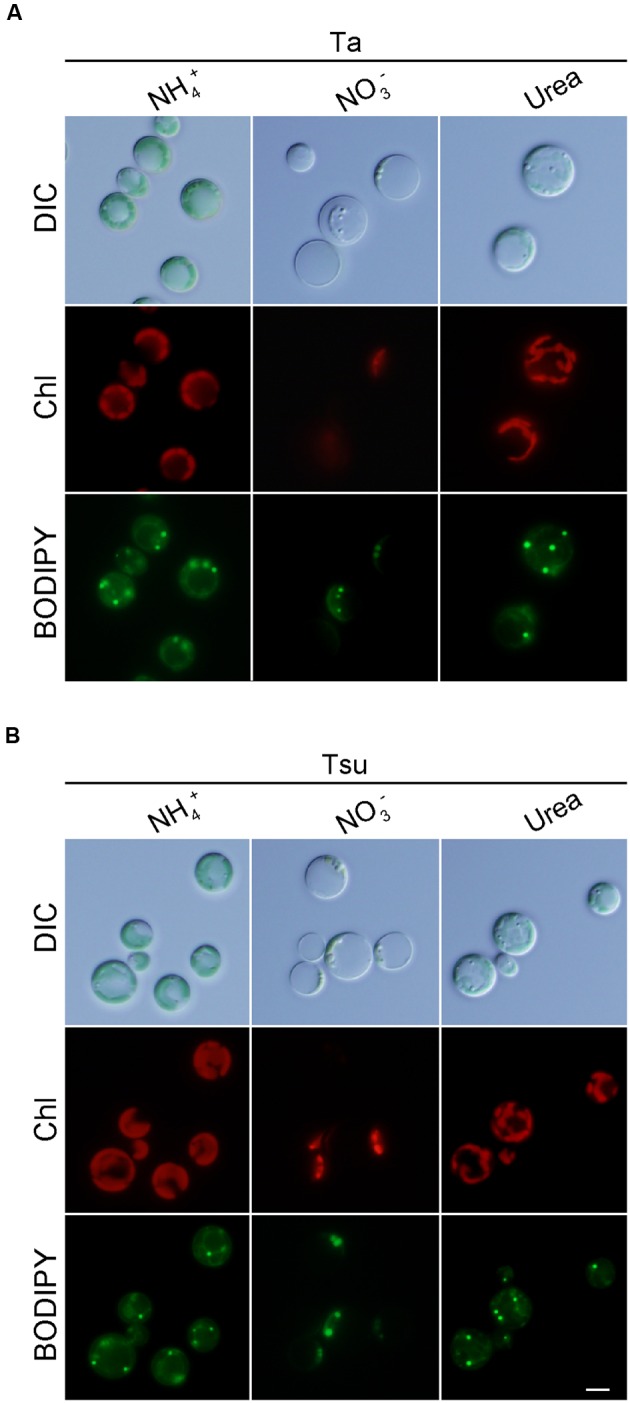
**Microscopic observation of *G. sulphuraria* 074G cells in media derived from acidic hot spring waters with different nitrogen sources. (A,B)** Micrographs of cells that were cultured in Ta **(A)** or Tsu **(B)** supplemented with different nitrogen sources. Cells cultured in the synthetic MA medium (OD_750_ of 1.0–2.0) were collected by centrifugation, resuspended into the respective media to give an OD_750_ of 1.0 and then cultured for 14 days. Images obtained by differential interference contrast microscopy (DIC), autofluorescence of chloroplasts (Chl) and BODIPY staining (BODIPY) are shown. Scale bar = 5 μm.

Cells also grew in the spring water media supplemented with urea (Ta+urea and Tsu+urea) (**Figure 3**). However, the growth rate in the media with the urea was lower than the respective spring water media with NH_4_^+^ (**Figures 3A,B,E**). In addition, the cellular chlorophyll and phycocyanin contents were lower than in the media supplemented with NH_4_^+^ (**Figure 3F**). The size of the chloroplasts was decreased and the appearance of the cells under microscopy was intermediate between that of the cells in the nitrogen-rich and nitrogen-limited/depleted media (**Figure 4**). Thus, urea is not suitable as a sole nitrogen source for *G. sulphuraria*, at least when cultured in the respective spring water media.

In contrast to the above media, cells did not grow in spring water media supplemented with NO_3_^-^ (Ta + NO_3_^-^ and Tsu + NO_3_^-^) (**Figures 3A–D**). Although the OD_750_ value slightly increased just after the inoculation from synthetic MA medium (**Figures 3A,B**), the cellular chlorophyll and phycocyanin contents (**Figure 3F**), size of chloroplasts and red autofluorescence (**Figure 4**) decreased, as in the case of the nitrogen-depleted MA medium. These results suggest that *G. sulphuraria* is not able to assimilate NO_3_^-^ under these culture conditions in the hot spring waters.

We also compared lipid droplet formation in the spring water media supplemented with NH_4_^+^, NO_3_^-^ or urea. However, there was a slight difference among the three media (**Figure 4**).

### Growth of *Pseudochlorella* sp. YKT1 in Media Derived from Acidic Hot Spring Water

**FIGURE 5:**
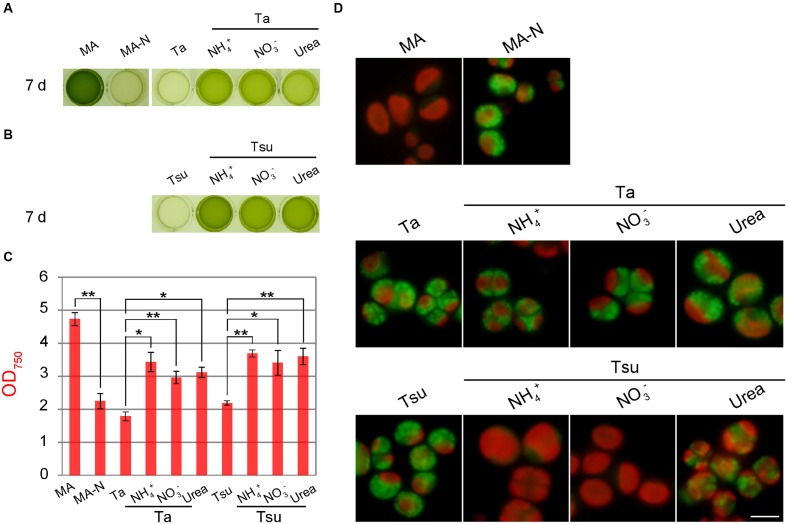
**Growth of *Pseudochlorella* sp. YKT1 cells in MA, MA-N and media derived from acidic hot spring waters supplemented with different nitrogen sources (pH 2.0)**. Cells cultured in MA (OD_750_ of 1.0–2.0) were collected by centrifugation, gently resuspended into the respective media to give an OD_750_ of 1.0 and then cultured for 7 days. **(A, B)** Photographs of the cultures in a 24-well plate at 7 days after inoculation. **(C)** OD_750_ of cultures at 7 days after inoculation. The bar indicates the standard deviation of three independent experiments. Significance was calculated by *t*-test. ^∗^Statistically significant differences at *P* < 0.05; ^∗∗^statistically significant differences at *P* < 0.01. **(D)** Micrographs of cells that were cultured in the respective media for 7 days. Scale bar = 5 μm.

In order to test whether the acidophilic green alga *P*. sp. YKT1 grows and accumulates lipid droplets in the spring water media, cells were cultured in a 24-well plate in which each well contained 1 mL of medium (**Figure 5**). The spring water media were adjusted to pH 2.0 with KOH and then filter-sterilized, because *P.* sp. YKT1 does not grow under extremely acidic conditions (pH < 2.0) (Hirooka et al., 2014).

When cells were inoculated into the respective media after being taken from the MA medium, cells did not grow in the spring water media without an additional nitrogen source (Ta and Tsu), as in the case of nitrogen-depleted synthetic medium (MA-N) (**Figures 5A,B**). In these media, the chloroplasts became smaller and many lipid droplets formed in the cells, as in the case of MA-N (**Figure 5D**), features which are indicative of nitrogen starvation. In contrast, when the spring water media were supplemented with either NH_4_^+^, NO_3_^-^ or urea (Ta + NH_4_^+^, Ta + NO_3_^-^, Ta + urea, Tsu + NH_4_^+^, Tsu + NO_3_^-^ and Tsu + urea), cells grew (**Figures 5A,B**) and reached 60–80% of the OD_750_ in synthetic MA medium 7 days after inoculation (**Figure 5C**). By means of gyration in Erlenmeyer flasks, cells entered into the stationary phase 3 weeks after the inoculation and the yield was 2.85 ± 0.13 g dry weight/L in MA, 2.49 ± 0.03 g dry weight/L in Ta + NH_4_^+^ and 2.95 ± 0.05 g dry weight/L in Tsu + NH_4_^+^, respectively.

In addition to the final algal biomass yield, there was no significant difference in the growth rate among the three nitrogen sources when compared in the same spring water media (**Figure 5C**). These results indicate that *P.* sp. YKT1 is able to utilize all three of these nitrogen sources. However, cells grew slightly better in Tsu-based media than in Ta-based media (**Figures 5A–C**), suggesting that more of a certain growth-limiting element, such as the phosphorus shown above (**Table 1**), is present in greater amounts in Tsu than Ta, or that Ta contains some growth-inhibiting element.

Although cells grew in all of the nitrogen-supplemented media, a high amount of lipid droplets appeared in the Ta-based media (**Figure 5D**). In addition, although fewer in number than in the Ta-based medium, lipid droplets were also formed in Tsu-urea (**Figure 5D**). In these cells with lipid droplets, the chloroplasts (observed as red autofluorescence) were smaller those in the cells in the other media, in which lipid droplets were scarcely detected (**Figure 5D**).

## Discussion

In this study, we have shown that sulfuric hot spring waters are applicable to the cultivation of acidophilic algae such as the red alga *G. sulphuraria* and the green alga *P*. sp. YKT1. It was also shown that, to culture these algae in the spring waters, an additional inorganic nitrogen source is required and in some cases, such as *G. sulphuraria*, the type of the nitrogen source (NH_4_^+^, NO_3_^-^, urea, or others) should be determined for the specific cultivation. The yield of the spring water cultivation is comparable to rich synthetic media in an appropriate spring water medium and a nitrogen source, being ∼2.73 g dry weight/L for *G. sulphuraria* (**Figure 3**) and ∼2.53 g dry weight/L for *P*. sp. YKT1.

It was shown that *G. sulphuraria* is not able to assimilate NO_3_^-^ in the spring waters tested (**Figure 3**). However, it has been suggested that *G. sulphuraria* should be able to grow with nitrate as the sole nitrogen source (Imamura et al., 2010), although the typical nitrate reductase gene has not been identified in the *G. sulphuraria* genome (Schönknecht et al., 2013). According to this suggestion, we confirmed that *G. sulphuraria* is able to grow in a synthetic nitrate medium (MA media containing NaNO_3_ instead of (NH_4_)_2_SO_4_ as the sole nitrogen source, pH 2.0). Thus, the preference in types of nitrogen sources depends on other factors in the media and it will be different for cultivation in spring water from cultivation in synthetic media.

In general, growth on ammonium shows a significant decrease in pH during the exponential phase, which causes growth inhibition due to the low buffering capacity of the medium (Eustance et al., 2013). Therefore, chemical buffers and pH regulators have been used to prevent a decrease in pH. However, *G. sulphuraria* was able to grow well without additional chemical buffers and pH controllers in this study. It is known that *G. sulphuraria* grows at a wide range of low pH values (pH 0.05 – 5.0) (Doemel and Brock, 1971). In addition, the pH value of Ta + NH_4_^+^ or Tsu + NH_4_^+^ changed little during cultivation of *G. sulphuraria* for 2 weeks from 1.15 to 1.08 and from 1.14 to 1.04, respectively, in this study. Thus, the pH value is relatively stable at low pH at least for cultivation of *G. sulphuraria*.

Recently, it was reported that the freshwater cyanobacterium *Synechocystis* sp. PCC 6803 is able to grow in seawater-based media with additional nitrogen and phosphorus sources. However, cells grew partially in the absence of additional phosphorus source (Iijima et al., 2015). These results are consistent with the general understanding that seawater is poor in phosphorous and phosphorous is a growth-limiting nutrient for algae in the ocean (Moore et al., 2013).

Phosphorus is an essential nutrient for all organisms and has made a major contribution to agricultural and industrial development. However, the phosphorus derived from phosphate rock is a non-renewable resource and it is estimated that current global reserves will be depleted in 50–100 years (Cordell et al., 2009). In this study, we found that the waters from sulfuric hot springs are rich in phosphate (**Table 1**), *G. sulphuraria* (**Figures 1–4**) and *P*. sp. YKT1 (**Figure 5**) grew well in them without any need of an additional phosphorus source. Thus, the fact of being rich in phosphate is a strong advantage of sulfuric hot spring water for algal cultivation.

Phycocyanin is used as a natural blue dye in industrial applications. In this study, the phycocyanin contents in *G. sulphuraria* cultured in spring water media were 84.12 ± 3.93 μg/mg dry weight in Ta + NH_4_^+^ and 107.42 ± 1.81 μg/mg dry weight in Tsu + NH_4_^+^ (**Figure 3F**). These are relatively lower than but still comparable to the level reported in the *Spirulina platensis* (148.3 μg/mg dry weight) is currently used in the phycocyanin produced for commercial use (Bhattacharya and Shivaprakash, 2005). At present, the application of phycocyanin is limited because of its sensitivity to temperature and pH (Sarada et al., 1999). It was recently shown that the phycocyanin in *G. sulphuraria* is more thermostable than that in *S. platensis* (Moon et al., 2014). Thus, phycocyanin from thermophilic red algae such as *G. sulphurari*a in combination with spring water media will reduce the production cost and expand the range of applications.

Many studies have shown that eukaryotic algae accumulate triacylglycerol in lipid droplets under nitrogen-depleted condition (Goncalves et al., 2016). Consistent with these previous studies, both *G. sulphuraria* and *P*. sp. YKT1 cells formed lipid droplets in the nitrogen-depleted synthetic MA medium and in spring water media lacking any additional nitrogen source (**Figures 2** and **5**). Under these nitrogen-starved conditions, cell growth ceases, as reported in other algae (**Figure 1**). However, we also observed lipid droplet formation in both *G. sulphuraria* and *P*. sp. YKT1 in the spring water media in which additional nitrogen, but not phosphate, was supplemented (i.e., *G. sulphuraria*, Ta + NH_4_^+^ and Tsu + NH_4_^+^ in **Figure 2**; *P*. sp. YKT1, Ta + NH_4_^+^, NO_3_^-^ or urea, and Tsu + urea; **Figure 5**), in which cells kept growing.

In *Chlamydomonas reinhardtii*, it was recently reported that phosphorus depletion substantially induces accumulation of lipid droplets in the cytosol while maintaining thylakoid membranes (Iwai et al., 2014). However, in this reported case, cell growth ceased, probably because of a complete depletion of phosphate from the medium. Although the acidic spring waters (Ta and Tsu) contained phosphorus at levels of a eutrophic lake, such a phosphorus level is still much lower than the level in a synthetic medium (**Table 1**). Thus, it is probable that the phosphate concentration of the sulfuric spring waters is sufficient for cellular growth, but induces lipid droplet formation. In the spring water media with an additional nitrogen source, *P*. sp. YKT1 poorly formed lipid droplets in Tsu-based media compared with Ta-based media (**Figure 5D**). One possible cause for this difference in the lipid droplet formation between Ta and Tsu is that the phosphate level in Ta is approximately one-third the level in Tsu (**Table 1**).

Unlike *G. sulphuraria*, *P*. sp. YKT1 cells form a high amount of lipid droplets under nitrogen-depleted condition and the cellular neutral lipid contents is elevated to ∼30% of the cellular dry weight (Hirooka et al., 2014). Thus, further investigation of acidophilic oleaginous algae and a determination of the appropriate conditions for lipid droplet formation in acidic spring water (or other acidic wastewater) will eventually lead to oil production while maintaining cellular growth at relatively low cost.

In a highly acidic environment, the number of species is limited compared to a neutrophilic environment. Thus, acidic wastewater from hot springs, mines and industries will provide a comparatively low cost alternative for open pond systems, since undesirable organisms will not be able to grow in it. In addition to searching for combinations of acidic water sources and useful acidophilic organisms, a reduction in the cost of the additional nitrogen source will facilitate the development of open pond algal culture systems.

## Author Contributions

Conceived and designed the experiments: SH, S-yM; performed the experiments: SH; analyzed the data: SH, S-yM; contributed reagents/materials/analysis tools: SH; wrote the paper: SH, S-yM.

## Conflict of Interest Statement

The authors declare that the research was conducted in the absence of any commercial or financial relationships that could be construed as a potential conflict of interest.
